# Intraductal papillary mucinous neoplasms of the pancreas and European guidelines: importance of the surgery type in the decision-making process

**DOI:** 10.1186/s12893-019-0580-y

**Published:** 2019-08-22

**Authors:** Etienne Buscail, Thomas Cauvin, Benjamin Fernandez, Camille Buscail, Marion Marty, Bruno Lapuyade, Clément Subtil, Jean-Philippe Adam, Véronique Vendrely, Sandrine Dabernat, Christophe Laurent, Laurence Chiche

**Affiliations:** 1 Department of Digestive Surgery, Haut Leveque Hospital, University of Bordeaux, Bordeaux, France; 2 Department of Epidemiology, EREN UMR INSERM INRA, University of Sorbonne Paris 13, Bobigny, France; 3 Department of Pathology, Haut Leveque Hospital, University of Bordeaux, Bordeaux, France; 4 Department of Radiology, Haut Leveque Hospital, University of Bordeaux, Bordeaux, France; 5 Department of Gastroenterology and Endoscopy, Haut Leveque Hospital, University of Bordeaux, Bordeaux, France; 6 INSERM 1035, University of Bordeaux, Bordeaux, France; 7 Department of Surgery, Haut Leveque Hospital, Bordeaux, France

**Keywords:** IPMN, Pancreatic resection, European guidelines, Post-operative morbidity

## Abstract

**Background:**

The European Consensus 2018 established a new algorithm with absolute and relative criteria for intraductal papillary mucinous neoplasms of the pancreas (IPMN) management. The aim of this study was to validate these criteria and analyse the outcomes in function of the surgical procedure and IPMN subtype.

**Methods:**

Clinical, radiological and surgical data (procedure, morbidity/mortality rates) of patients who underwent surgery for IPMN between 2007 and 2017. The predictive value of the different criteria was analysed.

**Results:**

124 patients (men 67%; mean age 65 years) underwent surgery for IPMN (*n* = 62 malignant tumours; 50%). Jaundice, cyst ≥4 cm and Wirsung duct size 5–9.9 mm or ≥ 10 mm were significantly associated with malignancy (4.77 < OR < 11.85 *p* < 0.0001). The positive predictive value of any isolated criterion ranged from 71 to 87%, whereas that of three relative criteria together reached 100%. The mortality and morbidity (grade III-IV complications according to the Dindo-Clavien classification) rates were 3 and 8%, respectively. Morbidity/mortality after duodenopancreatectomy and total pancreatectomy were significantly higher for benign IPMN (*p* = 0.01).

**Conclusion:**

Considering the morbidity associated with extended surgery, particularly for benign IPMN, the results of the present study suggest that high-risk surgery should be considered only in the presence of three relative criteria and including the surgery type in the decision-making algorithm.

**Electronic supplementary material:**

The online version of this article (10.1186/s12893-019-0580-y) contains supplementary material, which is available to authorized users.

## Background

Intraductal papillary mucinous neoplasm of the pancreas (IPMN) is characterized by adenomatous proliferation of the pancreatic duct epithelium that may involves the main pancreatic duct, the branch ducts, or both [1]. Accordingly, IPMNs are classified in three groups: main pancreatic duct (MD), branch duct (BD), and mixed tumours. IPMN malignant transformation occurs in 25–70% of cases, of which 15–43% are invasive, especially in the case of MD and mixed IPMN [2–4]. Surgical resection remains the best treatment to avoid this unfavourable outcome. However, two major problems remain: first, many BD IPMN (75–82%) will never progress to malignancy; and second, pancreatic surgery inherent morbidity is not negligible [2, 3]. Therefore, it is crucial to identify the patients who will actually need surgical resection.

Several guidelines have been established to define the surgery criteria, depending on the presence or not of “worrisome signs” and “high risk stigmata of malignancy” (i.e., high grade dysplasia and invasive carcinoma) [5–8]. In these algorithms, the patient’s performance status is taken into account to determine the surgery benefit/risk ratio. Recently, several European societies (i.e., the European Study Group on Cystic Tumours of the Pancreas, United European Gastroenterology, European Pancreatic Club, European-African Hepato-Pancreato-Biliary Association, European Digestive Surgery, and European Society of Gastrointestinal Endoscopy) published updated evidence-based guidelines for the management of pancreatic cystic neoplasms [9, 10].

In this study, we retrospectively analysed the clinical, imaging and pathological data of patients with IPMN who underwent surgery to evaluate: i) the diagnostic value of the relative and absolute European criteria or IPMN surgical resection; and ii) the post-operative morbidity and mortality rates.

## Methods

### Patients and inclusion criteria

Between January 2007 and December 2017, 720 patients underwent surgery for pancreatic adenocarcinoma or IPMN. Among them, all consecutive patients with BD, MD or mixed IPMN eligible for pancreatic surgery (i.e., resectable IPMN at imaging) and who underwent resection were included in the present study. All resected IPMN were histologically proven.

This study was conducted according the French rules (Bioethics law of November 2016 – research category number 3), and the Comité National Informatique et Liberté (French Data Protection Authority) recommendations for the anonymous extraction and treatment of data. According to these rules, due to the study retrospective nature, it was not necessary to obtain the patients’ informed consent for the analysis of their personal data. The study protocol conforms to the ethical guidelines of the 1975 Declaration of Helsinki (6th version, 2008) and was approved by the Bordeaux university hospital ethics committee (Direction recherche Clinique centre hospitalier universitaire de Bordeaux). This study followed the STROBE statement guidelines.

### Collected data

Since January 2005, all clinical, radiological and pathological data of patients undergoing pancreatic surgery at our centre are prospectively recorded in a dedicated database. We extracted the main clinical and radiological data, including most of the criteria retained by the recent European Consensus: age, sex, medical history, circumstances of diagnosis and symptoms related to IPMN, date of diagnosis, and biological data (including hepatic enzymes, blood lipase level), as well as the results of computerized tomodensitometry (CT) (helical triple-phase CT, each pancreatic section was effectively collimated into 3-mm sections at a pitch of 1.5), magnetic resonance cholangiopancreatography (MRCP) (1-T superconducting magnetic-resonance unit Magnetom Impact, Siemens, Erlangen, Germany, using a half-Fourier single-shot turbo spin-echo sequence), endoscopic ultrasound (EUS), and fine-needle aspiration biopsy (FNA) (Olympus, Hamburg, Germany). The collected imaging data were: maximal diameter of the MD and side branch duct cyst, tissue component/ thickening surrounding the MD or BD, and mural nodules with a size ≥5 mm.

According to the 2018 European guidelines, all absolute (tumour-related jaundice, presence of contrast-enhancing mural nodule ≥5 mm, solid mass, MD size ≥10 mm) and relative (rapid growth rate, MD size between 5 and 9.9 mm, cyst diameter ≥ 40 mm, new onset of diabetes, acute pancreatitis caused by IPMN) criteria for surgery were analysed, with the exception of positive cytology for malignant/high grade dysplasia, increased levels of CA 19.9 (≥37 UI/ml) and enhancing mural nodules < 5 mm in size due to too many missing data.

### Surgical data

Type of resection and postoperative complications were collected for patients who underwent surgery. All surgical indications were discussed and validated by our pancreas disease multidisciplinary group [5, 11]. In all cases, the surgical approach was surgical resection with appropriate lymphadenectomy, and frozen section analysis of the resection margins. The surgical approaches were classified as follow: distal pancreatectomy, pancreatoduodenectomy, total pancreatectomy, enucleation, and median pancreatectomy. Surgical morbidity was defined by the occurrence of grade III, IV or V postoperative complications, according to the classification by Dindo and Clavien [12]. Grade III includes complications that require surgical, endoscopic or radiological intervention, with or without general anaesthesia. Grade IV includes life-threatening complications that require management in an intensive care unit. Grade V complications cause postoperative death. Pancreatic fistula and post-operative haemorrhage were both defined according to the International Study Group of Pancreatic Surgery (ISGPS) classification [13, 14]. The macroscopic pathological analysis followed a standardized protocol in which the pancreas tissues were cut in serial sections that include the tumour up to the inked margins.

### Histopathological data

The resected tumour pancreatic tissue specimens were characterized and subtyped according to the Verona Consensus Meeting recommendations [1]. IPMN was classified in three subtypes: MD, BD and mixed. Following the 2010 World Health Organization criteria, high-grade dysplasia and invasive adenocarcinoma were considered malignant, whereas low grade dysplasia were considered benign IPMN [15]. The tumour cytological subtype was also reported (oncocytic, gastric, intestinal, hepatobiliary).

### Statistical analyses

Qualitative and quantitative data (Student’s-t, χ^2^, exact Fisher tests) were analysed using the GraphPad-Instat and GraphPad-Prism 6.0a software programs (GraphPad Software Inc. San Diego, CA, USA). To identify independent predictors of malignancy, univariate and multivariate logistic regression analyses were performed using logistic regression tests with the SAS software (version 9.4, SAS institute, Cary, NC, USA). A *p*-value < 0.05 was considered significant. Due to the retrospective design of the study a post-hoc analysis was performed to evaluate the power of the study. The power analysis was performed using G-power software 3.1.9.2.

## Results

### General characteristics, pre-operative imaging data and surgical procedures

**Fig. 1:**
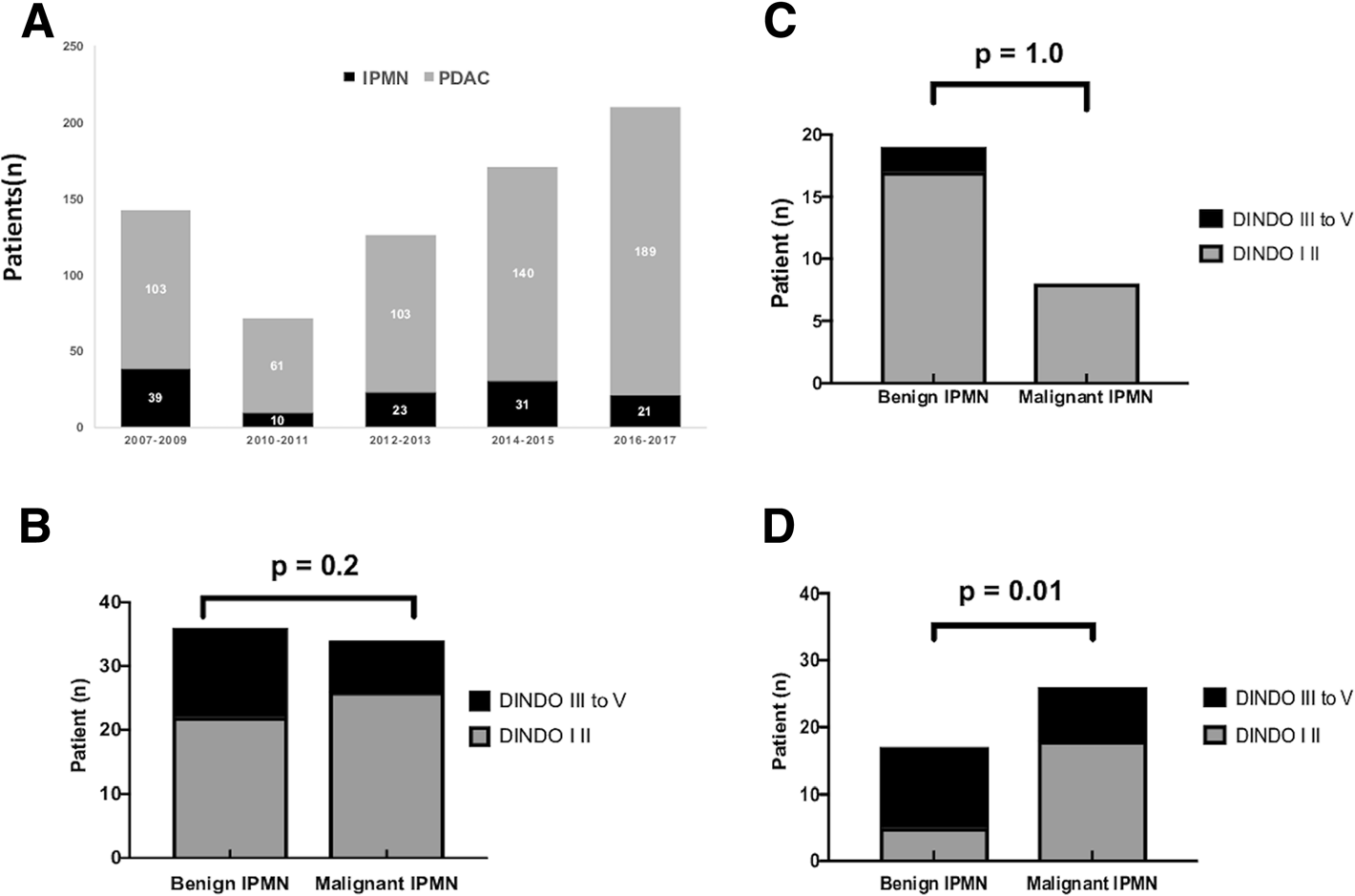
Number of pancreatectomies for IPMN between 2007 and 2017 and surgery complications. **a**: Number of pancreatic resection for pancreatic ductal adenocarcinoma (PDAC) (grey bars) and for IPMN (black bars) during the study period. **b**: Number of patients with post-surgery complications among the 124 patients who underwent pancreatectomy (all procedures) and subdivided according to the type of IPMN at the post-surgery histological analysis (*n* = 62 benign IPNM and *n* = 62 malignant IPMN). **c**: Comparison of post-surgery complications in the 49 patients who underwent distal pancreatectomy and subdivided according to type of IPMN at the post-surgery histological analysis (*n =* x benign IPNM and *n =* x malignant IPMN). **d**: Comparison of post-surgery complications in the 75 patients that underwent pancreaticoduodenectomy and total pancreatectomy and subdivided according to type of IPMN at the post-surgery histological analysis (*n* = 29 benign IPNM and *n* = 46 malignant IPMN). **b**-**d**: Grey bars, grade I-II complications; black bars, grade III to V complications, according to the Dindo-Clavien classification

In total, 124 consecutive patients were included (18% of all patients who underwent pancreatic resection in our department during the study period) (Fig. 1, panel A). The main characteristics and the imaging data at diagnosis and during the pre-surgery follow-up of these 124 patients and of the subgroup who had duodenopancreatectomy or total pancreatectomy (*n* = 75) are summarized in Table 1. The patients’ performance status was relatively good (American Society of Anaesthesiologists physical status: 1–2 for most of them), the most frequent symptoms were acute pancreatitis and abdominal pain (upper part, without hyperlipasemia), 36% of patients were asymptomatic, and 71% of patients had at least two different types of imaging exams. In Table 2 are detailed the indications for surgery, surgical procedures, post-operative complications, pathological findings and macroscopic subtyping (BD IPMN in 33 patients, MD IPMN in 46, and mixed form in 45; 73% of patients had MD or mixed IPMN). Table 3 shows the histological classification in term of malignancy, and the cytological subtyping depending on the anatomical involvement. Malignancy rate and histological subtype distribution were comparable in BD and MD/mixed IPMN samples. Comparison of the diagnostic performances of each imaging technique for recognizing BD and MD/mixed IPMN (Additional file 1: Table S1 and Additional file 2: Table S2) indicated that CT was more suitable for the diagnosis of BD IPMN, while MRCP was the most accurate imaging tool for the diagnosis of MD/mixed forms. EUS was slightly less accurate for both IPMN forms.

**Table 1:** Clinical characteristics at diagnosis and laboratory/imaging exams of all patients with IPMN who underwent surgery (*n* = 124) and of the subgroup who had duodenopancreatectomy or total pancreatectomy (*n* = 75)

Variables	All type of resection *N* = 124 *N (%)*	Whipple procedure and total pancreatectomy *N* = 75 *N (%)*	Distal pancreatectomy *N* = 45 *N* *(%)*	*P* *
Age *(median; range)*	66.1 *(67; 28–84)*	69.2 *(68; 53–80)*	63,8 *(66,5; 28–84)*	0.1
Men, *n (%)*	60 *(48)*	39 *(52)*	19 *(15)*	0.3
ASA score				
1	38 *(31)*	25 *(34)*	12 *(26)*	
2	81 *(65)*	48 *(64)*	30 *(66)*	
3	5 *(4)*	2 *(2.5)*	3 *(8)*	
4–5	0	0	0	0.4
First symptoms				
Acute pancreatitis	23 *(18.5)*	14 *(18)*	9 *(20)*	0.9
Jaundice	8 *(6.5)*	8 *(10.5)*	0	0.9
New onset of diabetes	8 *(6.5)*	8 *(10.5)*	0	0.9
Weight loss	8 *(6.5)*	6 *(8)*	2 *(4)*	0.7
Diarrhoea and/or steatorrhea	3 *(2.5)*	3 *(4)*	0	0.3
Signs of chronic pancreatitis	6 *(5)*	7 *(9.5)*	0	0.2
Abdominal pain	23 *(18.5)*	14 *(18)*	9 *(20)*	0.9
No symptom	45 *(36)*	22 *(29)*	20 *(45)*	0.1
Serum CA 19–9 (*n* = 33; 27%)		Serum CA 19–9 (realized *n* = 20; 27%)		
Normal	21 *(63)*	12 *(60)*	9 *(20)*	0.7
Elevated	12 *(37)*	8 *(40)*	4 *(9)*	
CT	106 *(86)*	65 *(86)*	41 *(90)*	0.5
MRCP	74 *(59)*	44 *(59)*	30 *(66)*	0.5
EUS	68 *(55)*	39 *(52)*	*25 (56)*	0.8
*(EUS-FNA)*	*(29; 43%)*	*(20; 51%)*	*(9; 20%)*	
At least two radiologic investigations	89 *(71)*	39 *(52)*	45 *(100)*	
MRCP + CT	57 *(46)*	28 *(37)*	29 *(64)*	
EUS + CT	56 *(45)*	33 (*44)*	23*(51)*	
EUS + MRCP	45 *(36)*	25 *(34)*	20 *(45)*	
EUS + CT + MRCP	34 *(27.5)*	20 *(27)*	14 *(31)*	

*ASA*: American Society of Anaesthesiology; *CT*-scan: computerized tomodensitometry; *MRCP*: magnetic resonance cholangiopancreatography; *EUS*: endoscopic ultrasound; *FNA*: fine needle aspiration

* t-test with Welch’s correction for age; fisher exact test for the other variables

**Table 2:** Indications for surgery, surgery type and complications, and pathology analysis results of the surgically removed IPMN

Variables	All resection types *N* = 124 *N (%)*	Whipple procedure and total pancreatectomy *N* = 75 *N (%)*	Distal pancreatectomy *N* = 45 *N (%)*	*p* Fisher exact test
Indication for surgery				
Symptoms	42 *(34):*	30 *(40):*	12 *(27)*	0,1
	*Acute pancreatitis N = 26*	*Acute pancreatitis N = 14*	*Acute pancreatitis N = 12*	
	*Jaundice N = 8*	*Jaundice N = 8*	*Jaundice N = 0*	
Imaging features	*Pain and/or new onset of diabetes N = 8*	*Pain and/or new onset of diabetes N = 8*	*Pain and/or new onset of diabetes N = 0*	
*(From Sendai and Fukuoka consensus)* Progression criteria	65 *(53)*	37 *(49.5)*	24 *(53)*	0.7
(Growth of 5 mm/year or increase in size of main pancreatic duct)(*)	17 *(13)*	8 *(11)*	9 *(20)*	0.1
Procedures				
Pancreatoduodenectomy	56 *(45)*			
Distal pancreatectomy	45 *(36)*			
Enucleation	3 *(2.5)*			
Total pancreatectomy	19 *(15)*			
Central pancreatectomy	1 *(0.5)*			
Post-operative complications				
Biochemical leak	39 *(31)*	15 *(27)*	20 *(44)*	
Grade B pancreatic fistula	6 *(5)*	5 *(9)*	1 *(2)*	
Grade C pancreatic fistula	5 *(2.5)*	5 *(9)*	0	
Post-operative haemorrhage (**)	6 *(5)*	4 *(5.5)*	2 *(4)*	
Dindo-Clavien III-IV	15 *(12)*	14 *(18)*	1 *(2)*	
Dindo-Clavien V	4 *(3)*	3 *(4)*	1 *(2)*	
Overall morbidity (***)	36 (29)	*31 (41)*	*5 (12)*	0.0001
Pathology: Macroscopic				
Branch duct	33 *(26.5)*	23 *(31)*	6 *(13)*	
Main duct	46 *(37)*	27 *(36)*	19 *(43)*	0.01
Mixed	45 *(36.5)*	25 *(34)*	20 *(44)*	(Branch duct vs Main duct and mixed)
Pathology: Microscopic				
Low grade dysplasia	51 *(41)*	20 *(26)*	28 *(62)*	
Intermediate dysplasia	11 *(9)*	9 *(12)*	0	
High grade dysplasia	25 *(20)*	18 *(24)*	7 *(14)*	0.01
Invasive carcinoma (****)	37 *(30)*	28 *(38)*	9 *(20)*	(Low and intermediate dysplasia vs high grade dysplasia/invasive carcinoma)
Pathology: Subtyping				
Gastric	15 *(12.5)*	6 *(8)*	9 *(20)*	
Intestinal	34 *(27)*	26 *(35)*	8 *(18)*	
Hepatobiliary	56 *(45)*	32 *(43)*	21 (*47)*	
Oncocytic	5 *(4)*	3 *(4)*	1 *(2)*	
Other	14 *(11.5)*	8 *(11)*	6 *(13)*	

* Follow up included clinical and CT scan evaluations at least every 6 months (median follow-up 12 month (range 6–17)

**: Grade A *n* = 2, Grade B *n* = 2, Grade C *n* = 2

***: Overall morbidity includes Grade B and C fistula, post-operative haemorrhage and Dindo-Clavien grade III-IV-V complication

***: Well-differentiated *n* = 16 *(43%)*; moderately differentiated *n* = 18 *(48%)*; poorly differentiated *n* = 3 *(9%)*

**Table 3:** Pathological characteristics of the 124 IPMNs classified according to their anatomical localization

Variables	Branch duct IPMN (*n* = 33–26.5%)	Main duct and mixed IPMN (*n* = 91–73.5%)	p*
Histology *(%)*			
*Low grade dysplasia*	21 *(63)*	41 *(45)*	0.21
*High grade dysplasia*	5 *(15)*	20 *(22)*	0.45
*Invasive carcinoma*	7 *(21)*	30 *(33)*	0.26
Total benign	21 *(63.5)*	41 *(45)*	
Total malignant	12 *(36.5)*	50 *(55)*	0.10
Cytological subtypes *(%)*			
Gastric	6 *(5)*	9 *(7)*	0.22
Intestinal	6 *(5)*	28 *(22.5)*	0.35
Hepatobiliary	19 *(15)*	37 *(30)*	0.10
Oncocytic	0	5 *(4)*	0.52
Other	2 *(1.5)*	12 *(9.5)*	0.34

*Fisher’s exact test

### Univariate and multivariate analyses of the absolute and relative European consensus criteria for predicting malignant IPMN

Analysis of the performance of each malignancy criterion retained by the European consensus using univariate and multivariate analyses (Table 4) showed that among the absolute criteria, jaundice and MD size ≥10 mm were significantly associated with malignancy (OR 11.85 and 7.52, respectively). Among the relative criteria, only cyst ≥4 cm and MD size 5 to 9.9 mm were significantly associated with malignancy (OR 5.61 and 4.77, respectively). Moreover, cyst size ≥3 cm, which was considered as a worrisome feature in the 2017 Fukuoka consensus guideline, was significantly associated with malignancy, but with a lower odds ratio (2.78). Finally, absence of symptoms, and MD size < 5 mm were associated with benign tumours (0.0005 < *p* < 0.0001 – relative risk 2.21 to 3.25).

**Table 4:** Univariate and multivariate analyses of the absolute and relative criteria from the 2018 European evidence-based guidelines as predictors of malignant IPMN in the 124 patients with IPMN who underwent pancreatectomy

Variables *Histology*	Univariate analysis			Multivariate analysis		
	OR	95% CI	*p*	OR	95% CI	*p*
Jaundice	7.76	(0.93–65.12)	*0.06*	**11.85**	**(1.17–119.79)**	***0.04***
*Benign (1–1.5%)*						
*Malignant (7–11%)*						
Acute pancreatitis	1.23	(0.50–3.01)	*0.65*	–		
*Benign (11–18%)*						
*Malignant (13–21%)*						
New onset of diabetes	0.31	(0.06–1.61)	*0.16*	0.17	(0.03–1.09)	*0.06*
*Benign (7–11%)*						
*Malignant (2–3%)*						
Cyst ≥4 cm	**4.87**	**(2.10–11.30)**	***0.0002***	**5.61**	**(2.02–15.5)**	***< 0.001***
*Benign (6–10%)*						
*Malignant (26–42%)*						
Cyst ≥3 cm (*)	**2.82**	**(1.34–5.96)**	***0.007***	**2.78**	**(1.80–6.54)**	***0.02***
*Benign (30–48%)*						
*Malignant (45–72.5%)*						
MD size 5–9.9 mm	**2.24**	**(1.08–4.64)**	***0.03***	**4.77**	**(1.78–12.77)**	***0.002***
*Benign (20–38.5%)*						
*Malignant (32–51.5%)*						
MD size ≥10 mm	**2.55**	**(1.15–5.64)**	***0.02***	**7.52**	**(2.47–22.89)**	***0.0004***
*Benign (13–21%)*						
*Malignant (25–40%)*						
Solid component	1.90	(0.81–4.45)	*0.14*	1.86	(0.67–5.20)	*0.24*
*Benign (11–18%)*						
*Malignant (18–29%)*						
Enhanced mural nodule > 5 mm	1.13	(0.43–3.01)	*0.80*	**–**		
*Benign (9–14.5%)*						
*Malignant (10–16%)*						
Rapid progression	0.75	(0.26–2.16)	*0.59*	**–**		
*Benign (9–14.5%)*						
*Malignant (7–1%)*						

*Total histology: Benign, n = 62 (50%), Malignant n = 62 (50%)*

*OR*: odds ratio; 95% *CI*: 95% confidence intervals; *MD*: main pancreatic duct

*: This risk factor was included as worrisome feature in the 2012 Fukuoka consensus guideline

Statistical significant results are in bold

### Specificity, sensitivity and predictive values of the absolute and relative criteria

Analysis of the predictive diagnostic values of the absolute and relative criteria of the recent European Consensus (Table 5) showed that all absolute criteria displayed high specificity (from 84 to 97%) and similar predictive diagnostic values, but low sensitivity. The positive predictive value (PPV) and negative predictive value (NPV) ranged from 53 to 71% and from 50 to 55%, respectively. Combining two or three absolute criteria did not increase these values.

**Table 5:** Diagnostic value of the absolute and relative criteria for pancreatic resection from the evidence-based European guidelines 2018 for predicting malignant IPMN. Analysis of the data for the 124 patients with IPMN who underwent surgery, and for the subgroup of 75 patient who duodenopancreatectomy or total pancreatectomy (PT) (shown in italics under each criterion)

Variables	Sensitivity (95% CI)	Specificity (95%CI)	PPV (95% CI)	NPV (95% CI)	Accuracy (95% CI)
Absolute criteria					
Solid component	29% (18–41)	87% (76–94)	69% (48–85)	55% (44–65)	58% (46–71)
*Whipple/Total PT*	*35%*	*76%*	*70%*	*43%*	*51%*
Jaundice	11% (4–21)	98% (91–99)	87% (47–99)	52% (43–61)	55% (44–68)
*Whipple/Total PT*	*15%*	97%	88%	42%	46%
Mural Nodule	16% (8–27)	85% (74–99)	53% (28–75)	50% (40–60)	50% (42–61)
*Whipple/Total PT*	*7%*	*80%*	*34%*	*35%*	*35%*
MD ≥ 10 mm	39% (26–51)	84% (73–91)	71% (52–84)	58% (46–68)	61% (48–79)
*Whipple/Total PT*	*48%*	*76%*	*76%*	*48%*	*59%*
At least two criteria *(n = 20)*	24% (14–36)	91% (84–97)	75% (59–91)	55% (44–64)	58% (44–67)
*Whipple/Total PT*	*31%*	*76%*	*67%*	*41%*	*48%*
At least three criteria *(n = 9)*	11% (4–21)	97% (88–96)	77% (40–97)	52% (42–61)	51% (40–60)
*Whipple/Total PT*	*16%*	*94%*	*78%*	*41%*	*46%*
Relative Criteria					
Cyst ≥ 4 cm (*)	42% (29–55)	90% (80–96)	81% (63–93)	61% (50–71)	66% (54–81)
*Whipple/Total PT*	*53%*	*76%*	*78%*	*50%*	*62%*
MD 5–9.9 mm	47% (34–60)	66% (53–77)	58% (43–72)	55% (43–67)	56% (44–71)
*Whipple/Total PT*	*35%*	*69%*	*64%*	*40%*	*48%*
Acute pancreatitis	21% (11–36)	82% (72–91)	54% (43–66)	51% (41–61)	52% (43–64)
*Whipple/Total PT*	*16%*	*80%*	*54%*	*38%*	*40%*
New onset of diabetes	3% (0.4–11)	88% (78–93)	22% (3–60)	48% (38–57)	46% (39–59)
*Whipple/Total PT*	*9%*	*83%*	*45%*	*37%*	*38%*
Rapid growth rate	1% (0.3–19)	83% (72–92)	37% (15–64)	48% (38–58)	47% (36–59)
*Whipple/Total PT*	*11%*	*90%*	*63%*	*39%*	*42%*
At least two criteria *(n = 30)*	30% (19–43)	82% (70–90)	63% (43–80)	54% (43–64)	56% (45–68)
*Whipple/Total PT*	*22%*	*63%*	*48%*	*34%*	*38%*
At least three criteria *(n = 9)*	14% (5–23)	100% (94–100)	100% (63–100)	54% (44–62)	57% (45–70)
*Whipple/Total PT*	*9%*	*100%*	*100%*	*41%*	*44%*

95% *CI*: 95% confidence interval

*PPV*: positive predictive value; *NPV*: negative predictive value

*: Cyst ≥3 cm (worrisome feature in the Fukuoka consensus guidelines 2012) had the following performances in the present study: sensitivity 66%, specificity 65%, *PPV* 65%, *NPV* 65% and accuracy 65%

Similarly, the sensitivity of all relative criteria was low, but their specificity ranged from 66 to 90%. The best predictive values were observed for cyst ≥4 cm (PPV 81%, NPV 66%), followed by MD size between 5 and 9.9 mm (PPV 58%, NPV 55%) and acute pancreatitis (PPV 54%, NPV 51%). Combining three relative criteria strongly increased these values, especially specificity and PPV that reached 100%.

The same analysis performed only in the subgroup of patients who underwent pancreaticoduodenectomy or total pancreatectomy gave similar or lower values compared with the entire cohort (Table 5).

The post-hoc analysis was based on our primary outcome (that is the improvement of the sensitivity against the usual criteria for diagnosis). Given a first-species risk of 5%, an increased sensitivity of 35% and our sample size (*N* = 124) and the power of our study was estimated at 97.3%.

### Post-operative complications

In this population, the overall surgical morbidity rate was 15% (*n* = 19), and the mortality rate was 3% (*n* = 4). Grade I and II and grade III-V complications were significantly more frequent in the subgroup with pancreaticoduodenectomy and total pancreatectomy than in the whole population (*p* < 0.0001, relative risk 6.03) (Table 2). Analysis of the post-operative complications depending on the final histology (benign versus malignant IPMN) did not find any difference between benign and malignant IPMN in the whole population (*n* = 124) (Fig. 1, panel B) and in the subgroup with distal pancreatectomy (Fig. 1, panel C). Conversely, in the subgroup with pancreaticoduodenectomy and total pancreatectomy (*n* = 75), complications were significantly more frequent in patients with benign IPMN than with malignant IPMN (*p* = 0.01) (Fig. 1, panel D, and Additional file 3: Table S3).

## Discussion

IPMN have a significant potential for malignancy, particularly the MD and mixed forms (50%) and to a lesser extent the BD forms (10 to 15%) [3, 16–19]. Decision-making in IPMN is a real challenge [20], because surgery is the only curative treatment of malignant IPMN. Therefore, for patients with IPMN, it is always necessary: i) to collect all information that might help to predict the risk of malignancy; ii) to choose the type of surgical procedure, depending on the IPMN location; and iii) to weigh the benefit/risk ratio of pancreatectomy.

The Fukuoka consensus conference of 2006, revised in 2017, established some baseline clinical and radiological criteria, especially in terms of tumour size and associated lesions (lymphadenopathy), to guide the decision concerning the patient management (surveillance or surgical treatment). This consensus separated criteria in “worrisome” and “high-risk stigmata” that allow or not the surgical option [6]. These criteria, complemented by the European, American Gastroenterological Association and American College of Gastroenterology guidelines are an important decision-making aid [7–10].

The recent update of the European consensus, which is based on evidence-based medicine, describes the surgical indication criteria (relative and absolute) for operable patients [10]. In the absence of these criteria, surveillance (intensive or not) is recommended. This issue is becoming important because IPMN are now more frequently identified. For instance, in the present study, pancreatic resection for IPMN accounted for 18% of all pancreatic surgeries, and the indication for surgery was recurrent pancreatitis or suspicion of malignancy [21–23]. Eventually 50% of resected tumours were benign, suggesting that surveillance could have been a reasonable option for some of these patients. Moreover, morbidity and mortality rates in our series were not negligible (15%). This represents an acceptable risk in the case of malignant disease, but debatable for benign disease.

We performed a retrospective analysis of all consecutive patients with IPMN who underwent surgery in our centre with the aim of evaluating the predictive value of the recent European consensus criteria [10] on the basis of the nature (benign vs malignant) of the resected IPMNs and the surgical morbidity/mortality.

First, analysis of the performance of the radiological and endoscopic criteria in our tertiary centre showed that they were in the same range as those previously published in pilot studies that display generally better performances [23–26]. MRCP and CT showed good performances to predict malignancies, whereas the results for EUS were slightly lower. This highlights the importance of combining complementary radiological investigations for the identification of the IMPN subtype and of malignant criteria, as previously demonstrated [27–29].

Second, we validated most of the absolute criteria that are significantly associated with the presence of malignant IPMN, with PPV from 70 to 87%. However, we found that the combination of 2-3 absolute criteria does not provide any additional diagnostic value compared with each single criterion. Nevertheless, one absolute criterion should be considered as an indication of resection in operable patients.

Concerning the relative criteria, it seems that all criteria are not equally reliable. Two of them were significantly associated with malignancy with good predictive values (60 to 80%): cyst size ≥4 cm and Wirsung duct size between 5 and 10 mm. We found that cyst size ≥3 cm (previously chosen as cut-off value) was less sensitive, specific, and with lower predictive values than cyst size ≥4 cm, finally chosen by the EU guidelines. On the other hand, rapid growth of the lesion at imaging did not have a high diagnostic value in our study. This criterion was not included in the previous consensus conference, whereas it is an integral part of a recent Korean study on a large number of patients with longitudinal follow-up. The authors suggested that rapid growth can be a predictive criterion for malignancy, and recommended a strict imaging-based surveillance [30].

The main result of our analysis is that the combination of two or three relative criteria significantly increased their diagnostic values with specificity and PPV values of 100%. All these elements should be considered, particularly if the risk of surgery complications is high.

However, due to the retrospective nature of our study it was difficult to take several c European consensus criteria into account in a formal way such as: assessing rapid growth of IPMN during the first 6 months after diagnosis, high grade dysplasia or malignant cytology, regular assessment of serum CA 19–9 level.

Our mortality rate was similar to that of other high volume centres [31]. We found, as expected, that extended pancreatic surgery (pancreaticoduodenectomy and total pancreatectomy) is associated with higher morbidity. Therefore, decision-making should be very rigorous and evidence-based, especially when more extended surgery is planned. We also found that morbidity after extended surgery was higher in patients with benign IPMN. This can be explained by the fact that in benign IPMN, the soft consistency of pancreas and the absence of dilation of the MD increase the risk of fistulae [32]. For the first time, the 2018 European guidelines included the patient’s clinical condition and age in the decision-making balance; however, they do not consider the surgery type (and therefore the IPMN localization/diffusion) and the associated risks. Indeed, they recommend IPMN management in specialized high-volume centres; however, even in such centres.

For this reason, we propose that the surgery type should be an important criterion in the decision-making process. When the decision of extended or risky surgery is based only on relative criteria, we think that at least two or more should be present, or the EUS should be repeated by an expert and associated with FNA.

It would have been interesting to have a control group mainly composed of patients without any relative or absolute criteria. However, our series is a surgical retrospective one and this may be a selection bias: indeed patients (otherwise operable for pancreatic targeting) have been referred to our tertiary referral surgical center because of IPMN with symptoms and/or at least a relative or absolute resection criterion. Moreover, the rate of malignant IPMN in the present work is of 50%, which is significantly higher than observed in “watch and wait attitude” series (the cumulative incidence of malignancy at 5 and 10 years in series including patients with low-risk IPMN is 3.5 and 7.7% respectively) [33]. Another limitation of our is that the inclusion period is 10 years during which the resection criteria have evolved (i.e. Sendai consensus then Fukuoka consensus 2012–2017 [5, 6, 11]).

## Conclusion

Our retrospective analysis of the performance of the absolute and relative criteria for IPMN malignancy shows that jaundice, cyst(s) ≥4 cm, and Wirsung duct diameter ≥ 10 mm are the most predictive absolute criteria of malignancy. Conversely, the diagnostic value of each isolated relative criterion is poor, and combining three relative criteria appears more relevant.

Considering the mortality and high morbidity rates of extended surgery (pancreaticoduodenectomy and total pancreatectomy) particularly in patients with benign IPMN, we suggest integrating the type of surgical procedure in the decision-making algorithm for IPMN management. A prospective study integrating the morbidity of the surgical procedure would validate this decision-making algorithm.

## Additional files

Additional file 1: **Table S1.** Diagnostic value of CT, MRCP and EUS for the preoperative diagnosis of IPMP subtypes in 124 patients who underwent surgical resection (DOCX 69 kb)

Additional file 2: **Table S2.** Distribution of IMPN subtypes and malignancy depending of the pre-operative presence of relative and absolute criteria for resection according the European guidelines 2018 (DOCX 14 kb)

Additional file 3: **Table S3.** Surgery data and post-surgery complications for the 75 patients with IPMN who underwent duodenopancreatectomy (Whipple procedure) or total pancreatectomy. Data are shown for patients with benign (low-intermediate dysplasia) and malignant (high grade dysplasia –invasive carcinoma) IPMN (post-surgery pathology) (DOCX 56 kb)

## Data Availability

From the corresponding author on reasonable request.

## Abbreviations

BD: Branch duct
CT: Computerized tomodensitometry
EUS: Endoscopic ultrasound
FNA: Fine-needle aspiration biopsy
IPMN: Intraductal papillary mucinous neoplasm
MD: Main pancreatic duct
MRCP: Magnetic resonance cholangiopancreatography
NPV: Negative predictive value
PPV: Positive predictive value
