# Supplementary material for: Intraductal papillary mucinous neoplasms of the pancreas and European guidelines: importance of the surgery type in the decision-making process
Source: BMC Surg. 2019 Aug 22;19:115. doi: 10.1186/s12893-019-0580-y (PMC6704670; doi:10.1186/s12893-019-0580-y)
Supplement: Supplementary file 1 — Table S1. Diagnostic value of CT, MRCP and EUS for the preoperative diagnosis of IPMP subtypes in 124 patients who underwent surgical resection (DOCX 69 kb) [file 12893_2019_580_MOESM1_ESM.docx]

**Supplemental Table 1:** Diagnostic value of CT, MRCP and EUS for the preoperative diagnosis of IPMP subtypes in 124 patients who underwent surgical resection

| **Variables** | **Sensitivity**  (95% CI) | **Specificity**  (95% CI) | **PPV**  (95% CI) | **NPV**  (95% CI) | **Accuracy**  (95% CI) |
| --- | --- | --- | --- | --- | --- |
| **CT**  **BD**  **MD/mixed** | 52% (32 - 70)  75% (63 - 85) | 84% (68 - 94)  45% (27 - 64) | 71% (48 - 80)  74% (61 - 84) | 69% (53 - 82)  47% (28 - 66) | 70% (54 - 83)  65% (49 - 78) |
| **MRCP**  **BD**  **MD/mixed** | 61% (40 - 78)  82% (69 - 91) | 65% (44 - 83)  70% (47 - 87) | 65% (44 - 83)  86% (73 - 94) | 61% (40 - 78)  64% (42 - 82) | 63% (42 - 81)  78% (53 - 91) |
| **EUS**  **BD**  **MD/mixed** | 55 % (39 - 70)  65 % (50 - 78) | 62% (38 - 82)  68% (43 - 87) | 74% (55 - 88)  83% (67 - 94) | 41% (24 - 59)  45% (26 - 64) | 57% (41 - 77)  66% (45 - 90) |

CT: computerized tomodensitometry; MRCP: magnetic resonance cholangiopancreatography; EUS: endoscopic ultrasound; BD: branch duct IPMN; MD: main duct IPMN; PPV: positive predictive value; NPV: negative predictive value; 95% CI: 95% confidence interval.
