# Supplementary material for: Intraductal papillary mucinous neoplasms of the pancreas and European guidelines: importance of the surgery type in the decision-making process
Source: BMC Surg. 2019 Aug 22;19:115. doi: 10.1186/s12893-019-0580-y (PMC6704670; doi:10.1186/s12893-019-0580-y)
Supplement: Supplementary file 2 — Table S2. Distribution of IMPN subtypes and malignancy depending of the pre-operative presence of relative and absolute criteria for resection according the European guidelines 2018 (DOCX 14 kb) [file 12893_2019_580_MOESM2_ESM.docx]

**Supplemental table 2.** Distribution of IMPN subtypes and malignancy depending of the pre-operative presence of relative and absolute criteria for resection according the European guidelines 2018

| Variables | Benign n=62 | Malignant (**) n=62 |
| --- | --- | --- |
| Relative indication for resection (*)  Total  Branch duct IPMN  Main duct and mixed | 18 (29%)  8 (13%)  10 (16%) | 6 (10%)  1 (2%)  5 (8%) |
| Absolute indication for resection (*)  Total  Branch duct IPMN  Main duct and mixed | 44 (71%)  30 (48%)  14 (23%) | 56 (90%)  31 (50%)  25 (40%) |
| Symptomatic IPMN  Worrisome or high-risk stigmata  Evolution of IPMN | 19 (31%)  33 (53%)  10 (16%) | 23 (37%)  32 (52%)  7 (11%) |

*: At least one criteria of the European Evidence-based Guidelines

**: High grade dysplasia and invasive carcinoma
