# Supplementary material for: Intraductal papillary mucinous neoplasms of the pancreas and European guidelines: importance of the surgery type in the decision-making process
Source: BMC Surg. 2019 Aug 22;19:115. doi: 10.1186/s12893-019-0580-y (PMC6704670; doi:10.1186/s12893-019-0580-y)
Supplement: Supplementary file 3 — Table S3. Surgery data and post-surgery complications for the 75 patients with IPMN who underwent duodenopancreatectomy (Whipple procedure) or total pancreatectomy. Data are shown for patients with benign (low-intermediate dysplasia) and malignant (high grade dysplasia –invasive carcinoma) IPMN (post-surgery pathology) (DOCX 56 kb) [file 12893_2019_580_MOESM3_ESM.docx]

**Supplemental Table 3:** Surgery data and post-surgery complications for the 75 patients with IPMN who underwent duodenopancreatectomy (Whipple procedure) or total pancreatectomy. Data are shown for patients with benign (low-intermediate dysplasia) and malignant (high grade dysplasia –invasive carcinoma) IPMN (post-surgery pathology)

|  | **Benign IPMN**  N = 29 *(%)* | **Malignant IPMN**  N = 46 *(%)* |
| --- | --- | --- |
| **Surgery duration** mean (min) *(median; range)* | 249 *(240; 140-360)* | 234 *(225; 100-500)* |
| **Pancreatic anastomosis (*)**  Pancreaticogastrostomy  Pancreticojejunostomy  No anastomosis | 7 *(12.5)*  10 *(18)*  3 *(5.5)* | 19 *(34)*  15 *(27)*  2 *(3.5)* |
| **Grade III/IV^(**)^** | 9 *(12)* | 5 *(6.5)* |
| **Grade V^(**)^** | 2 *(2.5)* | 1 *(1.5)* |
| **POPF**  Biochemical leak  Grade B  Grade C | 6 *(8)*  3 *(4)*  4 *(5.5)* | 9 *(12)*  2 (2.5)  1 *(1.5)* |
| **POPH**  Grade A/B  Grade C | 2 (7)  1 *(1.5)* | 0  1 *(1.5)* |

*: subgroup of patients with Whipple procedure n=36; **: according to the classification by Dindo and Clavien; POPF = post-operative pancreatic fistula; POPH: post-operative pancreatic haemorrhage.
